# History of Comparative Ophthalmics

**Published:** 1899-06

**Authors:** 

**Affiliations:** Stuttgart


### ABSTRACTS FROM FOREIGN JOURNALS.

### GERMAN.

Under the Direction of J. Preston Hoskins, Ph.D.,
Princeton, N. J.

History of Comparative Ophthalmics. By Königshöfer, Stuttgart.—Before the time of Christ no scientific or medicinal articles on diseases of the eye in animals are to be found. In Pliny (first century A.D.) two passages refer to this subject. In Book XI., Cap. 55, this author relates that some beasts of burden have eye troubles about the time of full moon; and a little further on, in the same chapter, he notes that cross-eyes never occur among animals. Pliny shows, further, a pretty accurate knowledge of the formation of the human eye. Galen wrote down some very accurate observations in regard to the anatomy of the human eye and that of the larger domestic animals. Vegelius Renatus (450 A.D.), in his *Arz Veterinaria*, describes some diseases of the eye of the horse—*e. g.*, the gray cataract (sufficio), paracentitis of the eye, moonblindness, and corneal cloudiness. From this time until well into the sixteenth century works on ophthalmics are totally lacking. The anatomy and physiology of the eye received new life through the works of Fallopia, Caserio, Stenon, Lenwenhoek, and Horius, who investigated the arrangement of the tear-organs and the microscopic construction of the eye.

Modern comparative ophthalmics begins in 1807, with the appearance of an article by the veterinarian Ammon, entitled "The Nature and Treatment of Inflammation in the Eye of the Horse." The work of Toggio, on *The Causes of Blindness in the Horse*, appeared in 1826, and was followed four years later by Leblanc's article on the "Eye-diseases of Domestic Animals, Especially the Horse." In Germany, Johann Friedrich Müller's *Manual of Veterinary Opththalmology for Veterinarians* appeared in 1844; but the new investigations of Graef and Helmholtz (1851), of Arlt and Donders, brought about a great modification of the views previously held. But veterinarians were rather slow to make use of the valuable materials gathered by these human ophthalmologists. It was Rudolf Berlin, in Stuttgart, who, with the establishment of a course in the ophthalmoscope in the veterinary school there, from 1875 on, introduced scientific ophthalmology to the students of veterinary medicine. To Berlin we owe a series of works in regard to the physical-optical construction of the eye of the horse. Berlin proved that the eyes of all domestic animals are hypermetropic; that they show an irregular astigmatism of the lens; that on the eye of the horse a regular corneal astigmatism is always to be found, etc. After these, his works on cataract, and operation for the same; on sinus thrombosis, with exophthalmos; on the operation for entropion; on the diagnosis of periodic inflammation, and others, appeared. Berlin's example exerted a good influence on other investigators. Bayer, in Vienna, published his *Comparative Ophthalmological Atlas*, and in his *Veterinary Chirurgie* elaborated ophthalmology very fully. Shortly afterward Möller published his *Ophthalmology for Veterinarians*. Schleich was Berlin's succsssor in Stuttgart, and he, too, is the author of a series of valuable anatomical and clinical works.

But still a whole series of anatomical and physiological questions are waiting to be cleared up. The theory of corneal inflammation in canine epilepsy and the differential diagnosis of periodic inflammation of the eye in the horse need to be sifted. The connection between eye-diseases and general conditions is in many cases obscure, and in regard to ophthalmic legislation much is to be desired. Comparative ophthalmology is, therefore, a territory where the investigator can look for good returns.—*Deut. Thierärztliche Wochenschrift*, June 11, 1898.

